# Bipotential mouse embryonic liver (BMEL) cells spontaneously express Pdx1 and Ngn3 but do not undergo further pancreatic differentiation upon Hes1 down-regulation

**DOI:** 10.1186/1756-0500-1-136

**Published:** 2008-12-24

**Authors:** Juliette Cuvelier Delisle, Lionel Martignat, Jean-Marie Bach, Steffi Bösch, Vanessa Louzier

**Affiliations:** 1 Immuno-endocrinology Unit 707, National Veterinary School, F-44300 Nantes, France; 2 Immuno-endocrinology Unit 707, French National Institute for Agricultural Research, F-44300 Nantes, France; 3 Immuno-endocrinology Unit 707, University, F-44000 Nantes, France; 4 ENVN, UMR 707 IECM, Atlanpôle La Chantrerie, 44307 Nantes cedex 3, France

## Abstract

**Background:**

Liver-to-pancreas conversion offers new possibilities for β-cell engineering for type 1 diabetes therapy. Among conceivable sources of liver cells, we focused on BMEL cells. These untransformed mouse embryonic liver cells have been reproducibly isolated from different inbred mice strains and have the potential to differentiate into hepatocytes and cholangiocytes *in vitro *and *in vivo*.

**Findings:**

Strikingly, we find here that adherent BMEL cells display functional similarities with multipotent pancreatic precursor cells, namely Pdx1 and Ngn3 expression, and further express *Hnf6 *in floating aggregate culture. Hes1, a direct repressor of Ngn3 and pancreatic endocrine commitment, is expressed in adherent BMEL cells and decreases with time in aggregate culture. However, Hes1 decrease fails to initiate activation of late-stage pancreatic endocrine transcription factors.

**Conclusion:**

Here we report that BMEL cells present features of pancreatic endocrine progenitor cells. In the field of diabetes research, BMEL cells are of potential interest for the study of inductive signals critical for in vitro β-cell maturation in-liver-to-pancreas conversion.

## Background

Diabetes results from the autoimmune destruction of pancreatic β-cells. The last years have witnessed major advances in engineering renewable supplies of β-cells through a combination of extrinsic cues and intrinsic reprogramming of hepatic stem cells. During embryogenesis, liver and pancreas arise from adjacent regions of the anterior foregut endoderm [1] and mutual plasticity between the liver and pancreas in the adult occurs in various pathological and experimental settings [2]. Lineage tracing studies have defined pancreatic master genes determining specification and maturation of the pancreatic endocrine cell lineage, namely pancreatic and duodenal homeobox-1 protein (Pdx1), bHLH proteins pancreatic transcription factor 1a (Ptf1a/P48) and neurogenin 3 (Ngn3) [3,4]. Conversely, loss of function and misexpression experiments demonstrate that the Notch pathway plays a pivotal role in pancreatogenesis as it maintains an undifferentiated progenitor population. Hairy enhancer and split 1 (Hes1) acts as a key downstream effector of the Notch pathway by repressing pancreatic endocrine cell specification via direct Ngn3 down-regulation [5]. Hes1 repression promotes conversion of biliary cells to pancreatic cells in developing tissues [6]. Although earlier findings demonstrate the feasibility of *in vitro *and *in vivo *liver-to-pancreas conversion [7], appropriate allo- or autologuous cell sources need to be characterised. Bipotential mouse embryonic liver cells have been reproducibly isolated from mouse embryos of different strains. BMEL cells have the potential to differentiate into hepatocytes and cholangiocytes in specific 3-D culture systems *in vitro *or after engraftment in damaged livers in Alb-uPA/SCID mice *in vivo *[8]. In the present study, we seek for the expression of pancreatic progenitor markers and Hes1 expression in BMEL stem cells in different culture modes.

## Methods

### Adherent BMEL cell culture

BMEL 9A1 cells, kindly provided by M. Weiss [8], were grown in RPMI 1640 (Invitrogen, Cergy-Potoise, France) supplemented with 10% foetal calf serum (Eurobio, Les Ulis, France), 50 ng/ml Epidermal Growth Factor (PeproTech, Levallois Perret, France), 30 ng/ml Insulin-like Growth Factor II (PeproTech), and 10 μg/ml human insulin (Roche, Meylan, France). Cells were cultured on dishes coated with Collagen I (Becton-Dickinson, Le Pont de Claix, France) in a humidified 5% CO_2 _atmosphere at 37°C. Cells were passaged every 3 to 4 days at 70.10^3 ^cells for a 100 mm dish, and never let sit at confluence.

### Aggregate BMEL cell culture

1,5.10^3 ^cells per well were seeded onto 96-well plates coated with Collagen I and cultured for two days as indicated previously. Cells were then dissociated with trypsin-EDTA (Eurobio) and 1.10^4 ^cells per well were seeded into 96-wells coated with poly(2-hydroxyethyl-methacrylate) (Sigma-Aldrich, Saint Quentin Fallavier, France) to which cells do not attach, but form floating aggregates within 24 hours. Aggregates were collected for analysis 4 days after seeding.

### mRNA isolation and RT-PCR reactions

mRNA was isolated with the Dynabeads mRNA Direct Kit (Invitrogen). First-strand cDNA was synthesized using M-MuLV reverse transcriptase (Promega, Charbonnières, France) and 400 nM random pentadecamer primers (Eurogentec, Liège, Belgique) [9]. cDNA was amplified by PCR over 35 cycles [94°C for 30 sec, 59 or 60°C for 30 sec, and 72°C for 40 sec] with RedTaq DNA Polymerase (Sigma-Aldrich) on a 9700 thermocycler (Applied Biosystems, Courtaboeuf, France) using the following primer pairs: β-*actin *(forward) 5'-AGCCATGTACGTAGCCATCC-3' (reverse) 5'-CTCTCAGCTGTGGTGGTGAA-3'; *hnf6 *(forward) 5'-CTGTGAAACTCCCCCAGGTA-3' (reverse) 5'-TCATCCCGCATAAGTGTGAA-3'; *ngn3 *(forward) 5'-GAGTTGGCACTCAGCAAACA-3' (reverse) 5'-TCTGAGTCAGTGCCCAGATG-3'; *pdx1 *(forward) 5'-CTGCGAGCTTCTGGAAAAAC-3' (reverse) 5'-CTGCTGGTCCGTATTGGAAC-3'; *neuroD *(forward) 5'-GAAAGCCCCCTAACTGACTGC-3' (reverse) 5'-GCACTTTGCAGCAATCTTAGCAAAA-3'; *nkx2.2 *(forward) 5'-GGTGGAGCGATTGGATAAGA-3' (reverse) 5'-TGCCATCAACCTTTTCATCA-3'; *nkx6.1 *(forward) 5'-AGTGATGCAGAGTCCGCCG (reverse) TCCTCATTCTCCGAAGTC-3'; *hes1 *(forward) 5'-CCTCTGAGCACAGAAAGTCATC-3' (reverse) 5'-TCCAGAATGTCTGCCTTCTC-3'; *insulin1 *(forward) 5'-CATCAGCAAGCAGGTYATTG-3' (reverse) 5'-CACTTGTGGGTCCTCCACTT-3'; *insulin2 *(forward) 5'-AGGACCCACAAGTGGCACA-3' (reverse) 5'-GAGGGGTAGGCTGGGTAGTG-3'; *ptf1a *(forward) 5'-GTAACCAGGCCCAGAAGGT-3' (reverse) 5'-CCTCTGGGGTCCACACTTTA-3'; *amylase *(forward) 5'-GGCCTTCTGGATCTTGCAC-3' (reverse) 5'-TCCTTGGGAGAACCATTTTG-3'.

Quantitative real-time RT-PCR was performed on 1 μl of cDNA with Prism 5700 sequence-detection system (Applied Biosystems). Cycling parameters were initiation at 50°C for 2 min and denaturation at 95°C for 10 min followed by 40 cycles of denaturation 95°C for 15 sec and annealing at 60°C for 1 min. Mouse *hes1 *(D16464) and *β-actin *(NM_007393) expression were amplified using 300 nM *hes1 *(forward) CCTCTGAGCACAGAAAGTCATC (reverse) GCATCCAAAATCAGTGTTTTCA, and *β-actin *(forward) CTCTTCCAGCCTTCCTTCCT (reverse) GGGCAGTGATCTCTTTCTGC primers in Power Sybr Green Master Mixture (Applied Biosystems), Relative real time PCR data were processed according to a standard curve based method [10] generating reference gene normalized expression values. Statistical comparisons between groups of treated versus untreated samples were performed using a non parametric Mann-Withney test.

### Immunocytochemistry

Ngn3, Pdx1, and Hes1 immunostaining was carried out on cytospins *of *40,000 BMEL cells after 4 days of adherent or suspension culture. Cells were fixed in 4% paraformaldehyde, permeabilized in PBS-Triton X-100 0,2%, and blocked in BPS, 1%BSA, 10% goat serum before incubation with primary antibodies in PBS -1% BSA. Primary antibodies used were: rabbit anti-Pdx1(1:3000), a gift from C. Wright, rabbit anti-Ngn3 (1:3000), a gift from M. German, and rabbit anti-Hes1 (1:60), a gift from T. Sudo. Secondary biotinylated goat anti-rabbit Ig antibody (Dako E0432) was used at 1:300 and detected with streptavidine-conjugated HRP (Dako, P397) and DAB substrate (Dako, SK-4100).

### Western blotting

After harvest with trypsin-EDTA, cells were washed with D-PBS and lysed in RIPA lysis buffer (Upstate, Euromedex, Mundolsheim, France) supplemented with protease inhibitors (Sigma-Aldrich, P8340). 50 μg of protein extract were fractionated by 8% SDS-PAGE and transferred to PVDF membrane (BioRAD, Marnes la Coquette, France). After blocking at room temperature for 2 h in TBS with 5% nonfat dry milk, the membranes were incubated for 1 h in TTBS containing 0,6 μg/ml polyclonal rabbit anti-mouse Hes1 antibody (kindly provided by T. Sudo) or polyclonal goat anti-mouse Pdx1 antibody (1:10000, kindly provided by C.V.E. Wright), and then for 1 h with TTBS containing secondary anti-rabbit (1:10000, Pierce, Perbio, Brebières, France) or anti-goat (1:10000, Dako) conjugates coupled to HRP. Antibody binding was visualized by ECL using Supersignal West Pico substrate (Pierce). Blots were stripped by 5 min incubation in 0,2 M NaOH, blocked for 1 h in TBS with 5% nonfat dry milk, and re-probed for 2 h in TTBS with primary anti-β-actin antibody (1:200, TEBU, Santa-Cruz) coupled to HRP. Anti-β-actin antibody binding was detected using ECL substrate as stated before. Band intensities were measured on a Chemidoc XRS system (BioRAD) and relative abundance of Hes1 protein normalized to β-actin was assessed by densitometry using Quantity One software (BioRAD).

## Results

### Characteristics of BMEL cells

**Figure 1:**
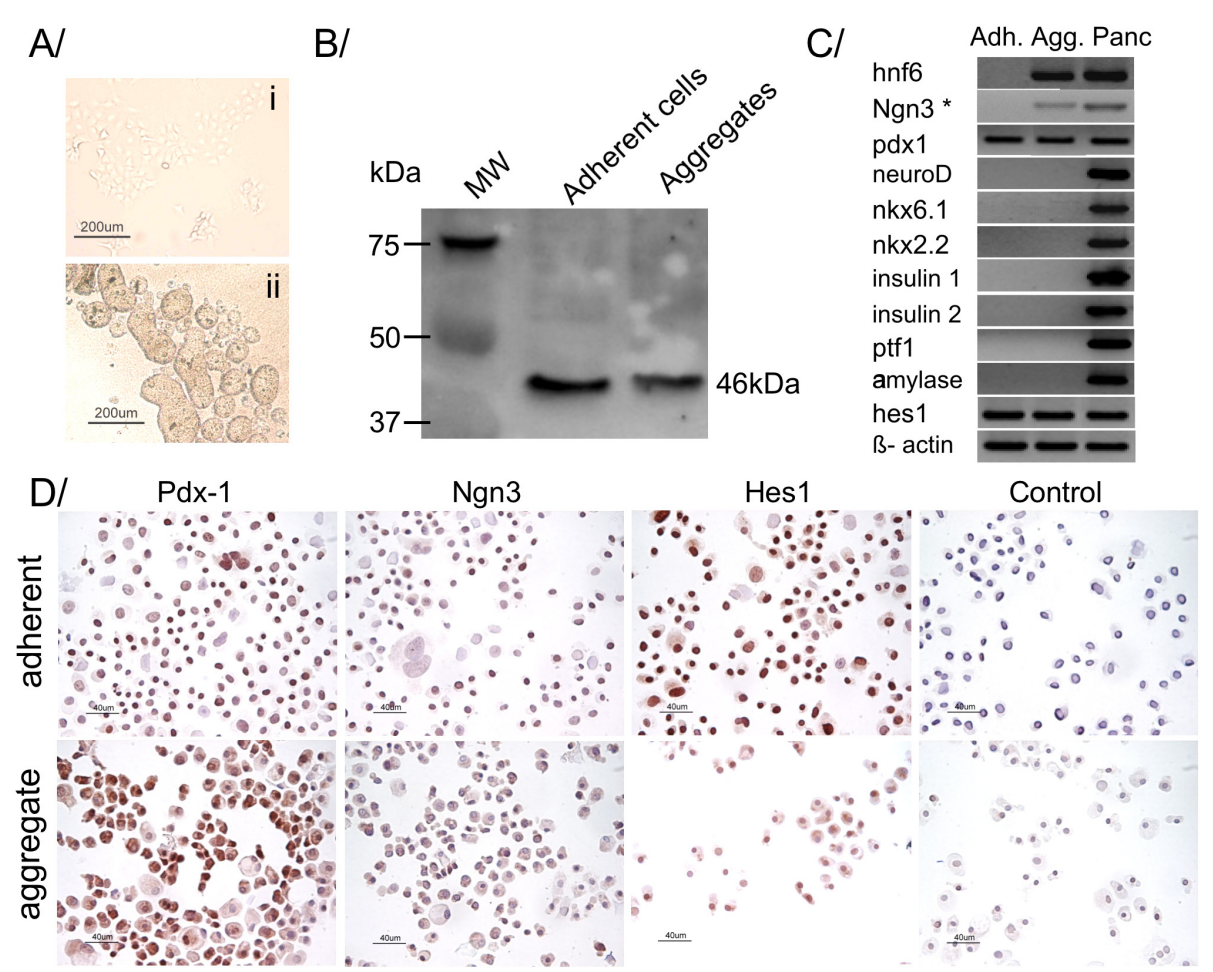
**BMEL characteristics in adherent vs aggregate culture**. (A) Morphology of BMEL cells grown for 4 days in (i) adherent or (ii) floating aggregate culture. (B) Western blot analysis of BMEL cells after four days of adherent or aggregate culture showing the expression of a 46kDa Pdx1 protein (n = 3). (C) RT-PCR profiles of adherent (Adh.) or aggregate (Agg) BMEL cells (n = 5). * Three out of five aggregate samples yielded positive *ngn3 *RT-PCR signals. cDNA from adult mouse pancreas (Panc) served as positive control for all genes except for Ngn3 for which a plasmid containing the *ngn3 *open reading frame was used as positive control. (D) Spontaneous Pdx1, Ngn3, and Hes1 expression located predominantly in the nuclei of adherent (top row) or aggregate (bottom row) BMEL cells. Scale bars = 40 μm.

BMEL cells can be propagated in an undifferentiated state in adherent culture on collagen-coated dishes. Here, we investigated pancreatic gene expression in BMEL cells after 4 days of culture. Adherent BMEL cells spontaneously expressed transcripts and protein of Pdx1 (Fig. 1), a regional endodermal marker found in pancreatic buds, at the origin of all pancreatic lineages [11,12]. Immunostaining also revealed a subpopulation of cells expressing Ngn3, which is considered to be the earliest marker specific to the endocrine lineage [4]. However, RT-PCR failed to detect *ngn3 *at the transcript level. At this stage, BMEL cells were devoid of Ptf1a and other markers of more advanced pancreatic commitment or mature β-cells. Suspension culture induced formation of floating aggregates and concurrent expression of hepatocyte nuclear factor 6 (*hnf6*), a factor known to activate *ngn3 *expression and to control the timing of the pancreas specification upstream of Pdx1 [13]. *Ngn3 *became detectable in 3 out of 5 samples at the transcript level and Ngn3 protein was present in a great majority of cells analysed by immuncytochemistry. Hairy enhancer and split 1 (*hes1*), a direct repressor of the *ngn3 *promoter, was detected by qualitative end-point RT-PCR in all samples regardless of culture mode even though immunocytochemistry experiments yielded less intense staining in cells of aggregate cultures.

### Hes1 expression decreased with time in BMEL aggregates

**Figure 2:**
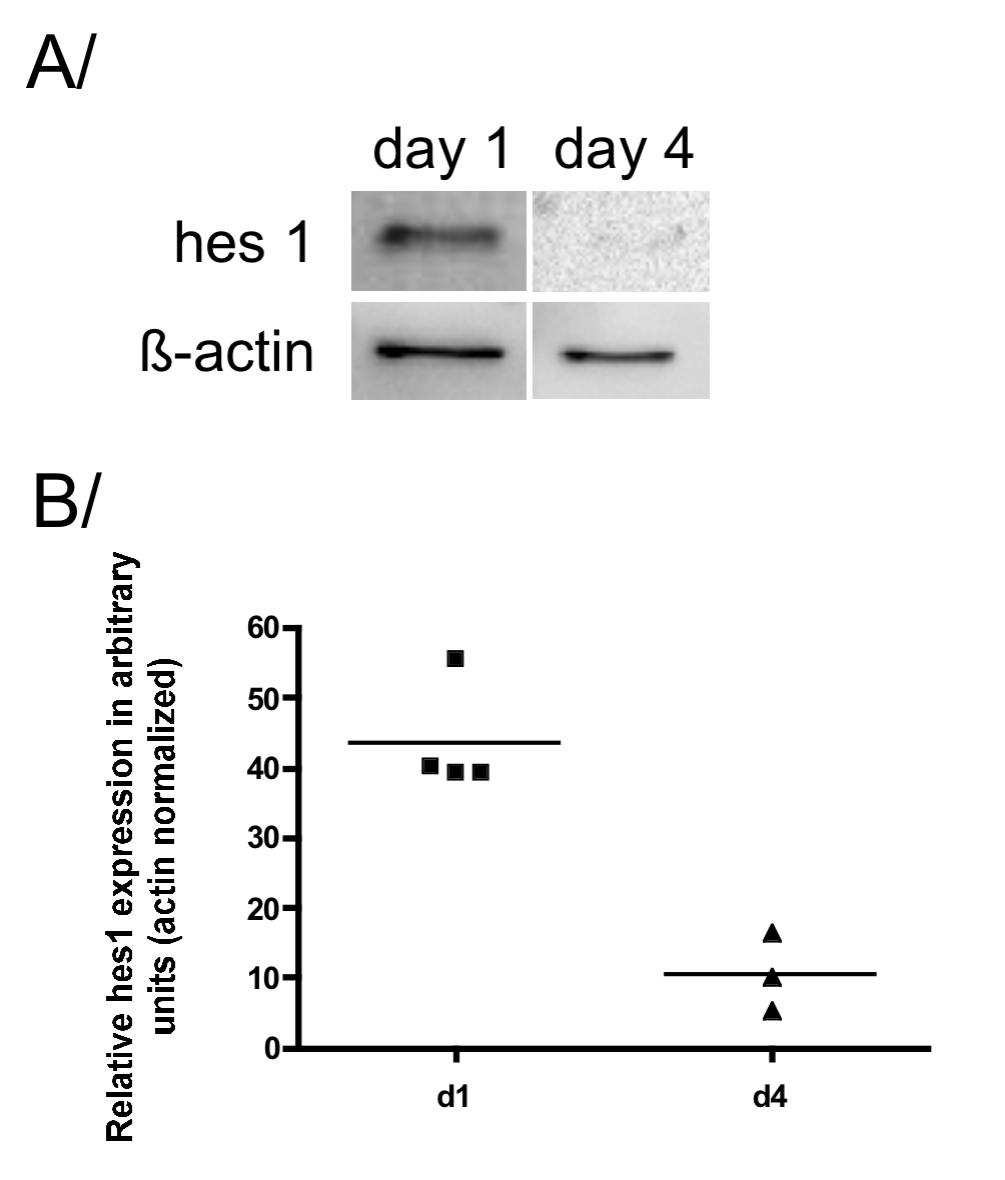
**Hes1 expression decreases with time in BMEL aggregates**. A/Hes1 western blotting (n = 4) on BMEL cells on day 1 and day 4 of aggregate culture. B/Real-time RT-qPCR analysis of Hes1 expression in BMEL cells on day 1 (n = 4) and day 4 (n = 3) of aggregate culture (p < 0,05). Each dot represents an independent experimental determination. Bars correspond to medians.

BMEL cells were grown for 1 or 4 days in aggregate culture and analyzed by Western blotting and real-time RT-qPCR. Sustained Hes1 expression at day 1 was nearly abolished at day 4 in aggregate culture with a good correspondence between the level of suppression at the protein and RNA levels (Fig. 2).

## Discussion

Liver-to-pancreas conversion offers new possibilities for β-cell engineering for cell therapy of diabetes [14]. Here we focus on untransformed BMEL cells, a new model of bipotential liver progenitor cells that have been reproducibly isolated from different inbred mice strains at 14 dpc. In adherent culture, these cells propagate with a high proliferation index in an undifferentiated state [8]. Strikingly, adherent BMEL cells in our hands also display functional similarities with multipotent pancreatic precursor cells, namely Pdx1 and Ngn3 expression, which does not occur in embryonic liver development. Indeed, Pdx1 is thought to act as a master regulatory gene, sufficient *per se *to distinguish liver and pancreas in normal development. No spontaneous Pdx1 expression in the hepatic lineage has been described yet during embryogenesis or in the adult liver in the absence of high glucose concentrations [15]. Western blot analysis reveals a 46 kDa endogenous Pdx1 protein, which is in accordance with the apparent weight of the active phosphorylated form of Pdx1 in earlier findings [16]. Ngn3, a true marker of endocrine commitment [4,17] is detected at the protein, but not at the transcript level in adherent cells. This apparent discrepancy may stem either from differences in the sensitivity of the techniques used here or from a greater stability of the protein. Following aggregate formation, BMEL cells further express Hnf6, and Ngn3 becomes detectable at the transcript as well as the protein level. These results fit with the idea that BMEL cells do not closely phenocopy bipotential hepatoblasts *in vivo*, and may present an enlarged differentiation potential. Immunocytochemistry results presented in Fig. 1 show that most BMEL cells evenly express Pdx1 and Ngn3 ruling out the idea that pancreatic progenitor-like cells form a sparse subpopulation. It is noteworthy that Ngn3-positive cells have a limited mitotic potential [18], opening the possibility for rapid overgrowth by less differentiated cells in culture. Apart from Pdx1, Hnf6, and Ngn3, BMEL cells do not express any of the downstream effectors of more advanced pancreatic commitment. Moreover, expression of pancreatic markers in aggregates remains unchanged following culture in high glucose medium, which is known to stimulate maturation of pancreatic progenitors (25 mM glucose, data not shown) [19]. Hes1, a direct repressor of Ngn3, is expressed in BMEL cells regardless of culture conditions, albeit quantitative real-time PCR shows a decrease with time in aggregate culture. Although Hes1 is expressed in intrahepatic bile duct cells at the neonatal stage, it is usually absent in hepatoblasts [20]. On the other hand, Hes1 is present in pancreatic progenitor cells where it acts as a dominant factor promoting self-renewal at the expense of β-cell differentiation [21]. Previous microarray data suggest that Notch signalling pathway is operative in maintaining BMEL cells in an undifferentiated state [22]. However, notch signalling disruption experiments failed to initiate activation of late-stage pancreatic transcription factors in BMEL aggregates (Additional_data_1). These results support the idea that factors beyond Hes1 expression, presumably the absence of Ptf1a, impede the extent of expression of Ngn3 and other factors of more advanced β-cell differentiation [23-25]. Indeed, lineage-tracing experiments have shown that endocrine i.e. Ngn3-positive cells derive from progenitors that express Ptf1a and, henceforth, Ngn3 and Ptf1a are usually co-expressed in pancreatic multipotent progenitors [3,23]. To summarize, here we report that BMEL cells display two stage-specific features of multipotent pancreatic progenitor cells, namely Pdx1 and Ngn3 expression. In the field of β-cell replacement research, BMEL cells are of potential interest for the study of inductive signals critical for β-cell maturation including positive selection of cells, trophic factors, or ectopic expression of pancreatic transcription factors like Ptf1a in complement to endogenous Pdx1 and Ngn3 expression.

## Competing interests

The authors declare that they have no competing interests.

## Authors' contributions

JC contributed to cell culture and RT-PCR analysis. VL carried out western blotting. SB performed real-time RT-PCR, immunostaining experiments, and drafted the manuscript. All authors contributed to the design of the study and interpretation of data. All authors read and approved the final manuscript.

## Supplementary Material

Additional file 1 **Notch pathway inhibition accelerates hes1 shut-down in BMEL aggregates**. Hes1 protein content after treatment with gamma-secretase-inhibitor, an inhibitor of the Notch signaling pathway. Click here for file
