# Supplementary material for: Bipotential mouse embryonic liver (BMEL) cells spontaneously express Pdx1 and Ngn3 but do not undergo further pancreatic differentiation upon Hes1 down-regulation
Source: BMC Res Notes. 2008 Dec 24;1:136. doi: 10.1186/1756-0500-1-136 (PMC2649931; doi:10.1186/1756-0500-1-136)
Supplement: Additional file 1 — Notch pathway inhibition accelerates hes1 shut-down in BMEL aggregates. Hes1 protein content after treatment with gamma-secretase-inhibitor, an inhibitor of the Notch signaling pathway. [file 1756-0500-1-136-S1.pdf]

Additional_ data_1.pdf

Notch pathway inhibition accelerates hes1 shut-down in BMEL aggregates

Hes1 protein content after treatment with gamma-secretase-inhibitor, an inhibitor of the Notch signaling pathway.

Additional Methods

Gamma-secretase inhibitor (GSI) cytotoxicity assay. Experiments were performed in collagen-coated microplates at $1,5.10^3$ cells per well. GSI (Sigma-Aldrich, S2188) was tested at concentrations of 10, 3.3, 1.1, and 0.33µM (n=2) or DMSO vehicle. Cell growth was estimated after 72h by a colorimetric assay based on conversion of a tetrazolium dye (MTT, Sigma) to a blue formazan product by living cells [1].

Treatment with gamma-secretase inhibitor (GSI). $1,5.10^3$ cells per well were seeded onto 96-well plates coated with Collagen I and cultured for two days in the presence of 3.3 µM of a peptidomimetic inhibitor of gamma-secretases (Sigma-Aldrich, S2188) or an equivalent amount of DMSO vehicle in the aforementioned medium (n=8). Cells were then detached with trypsin-EDTA (Eurobio) and 1.10^4 cells per well were seeded into 96-wells coated with poly(2-hydroxyethyl-methacrylate) (Sigma-Aldrich) in the presence of fresh 3.3 µM GSI or DMSO vehicle medium. Aggregates were collected for protein extraction on days 1, 2 and 4 following aggregate seeding and analyzed by Western blotting. mRNAs were isolated on day 4 of aggregate culture and analysed by RT-PCR.

Additional Results

Treatment with GSI accelerates Hes1 down-regulation in BMEL aggregates

GSI, a commonly used inhibitor of the gamma-secretase cleaving the Notch intracellular domain [2] inhibited BMEL cell growth by 70% after 72h of treatment at 3,3µM (data not shown). In comparison to untreated controls, Hes1 protein content was reduced nearly three- fold on day 1 (third day of GSI-treatment) and two-fold on day 2 (fourth day of GSI treatment) of aggregate culture as measured by densitometric analysis of western blots (Supplementary Fig.1). Hes1 protein content again fell close to detection level by day 4 (sixth day of GSI treatment). End-point RT-PCR analysis (n=8) on day 4 of GSI-treated aggregates yielded expression patterns of pancreatic molecular markers identical to untreated controls as depictured in Fig.1 in the main manuscript.

Supplementary Fig.1

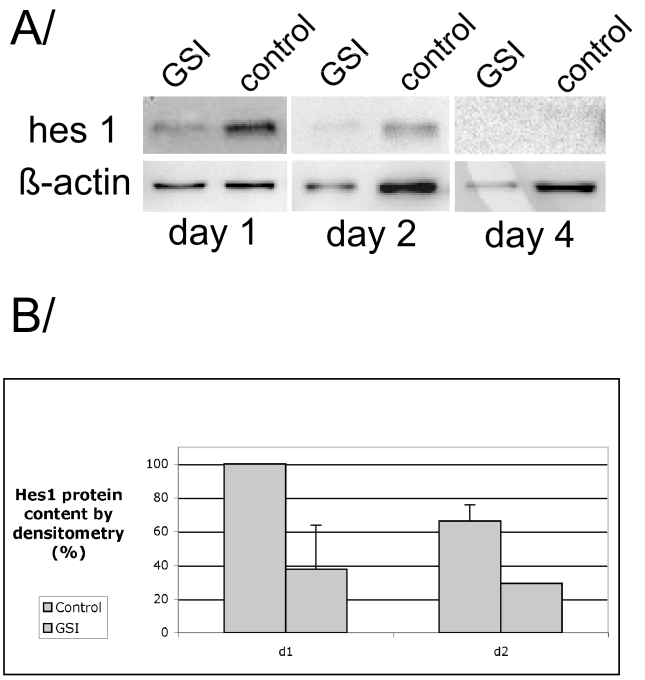

A/ Western blotting on day 1 (d1), day 2 (d2), and day 4 (d4) of BMEL cell

aggregates treated with gamma-secretase inhibitor (GSI), and B/ densitometric analysis of hes1 normalized to ß-actin on day 1 and day 2 western blots (n= 2 for d1 DMSO controls and GSI treated cells, and n=1 for d2 GSI treated cells).

1. Mosmann T: **Rapid colorimetric assay for cellular growth and survival: application to proliferation and cytotoxicity assays**. 1983, **65**(1-2):55-63.
2. Miralles F, Lamotte L, Couton D, Joshi RL: **Interplay between FGF10 and Notch signalling is required for the self-renewal of pancreatic progenitors**. *Int J Dev Biol* 2006, **50**(1):17-26.
